# Noradrenergic Source of Dopamine Assessed by Microdialysis in the Medial Prefrontal Cortex

**DOI:** 10.3389/fphar.2020.588160

**Published:** 2020-09-23

**Authors:** Paola Devoto, Claudia Sagheddu, Michele Santoni, Giovanna Flore, Pierluigi Saba, Marco Pistis, Gian Luigi Gessa

**Affiliations:** ^1^Section of Neuroscience and Clinical Pharmacology, Department of Biomedical Sciences, University of Cagliari, Cagliari, Italy; ^2^“Guy Everett” Laboratory, University of Cagliari, Cagliari, Italy; ^3^Department of Medical Sciences and Public Health, University of Cagliari, Cagliari, Italy; ^4^Section of Cagliari, Neuroscience Institute, National Research Council of Italy (CNR), Cagliari, Italy

**Keywords:** dopamine-norepinephrine interaction, noradrenergic denervation, norepinephrine transporter, D2 receptor drug, neuronal activity, ventral tegmental area

## Abstract

Previous results indicate that dopamine (DA) release in the medial prefrontal cortex (mPFC) is modified by α_2_ adrenoceptor- but not D2 DA receptor- agonists and antagonists, suggesting that DA measured by microdialysis in the mPFC originates from noradrenergic terminals. Accordingly, noradrenergic denervation was found to prevent α_2_-receptor-mediated rise and fall of extracellular DA induced by atipamezole and clonidine, respectively, in the mPFC. The present study was aimed to determine whether DA released by dopaminergic terminals in the mPFC is not detected by *in vivo* microdialysis because is readily taken up by norepinephrine transporter (NET). Accordingly, the D2-antagonist raclopride increased the electrical activity of DA neurons in the ventral tegmental area (VTA) and enhanced extracellular DOPAC but failed to modify DA in the mPFC. However, in rats whose NET was either inactivated by nisoxetine or eliminated by noradrenergic denervation, raclopride still elevated extracellular DOPAC and activated dopaminergic activity, but also increased DA. Conversely, the D2-receptor agonist quinpirole reduced DOPAC but failed to modify DA in the mPFC in control rats. However, in rats whose NET was eliminated by noradrenergic denervation or inhibited by locally perfused nisoxetine, quinpirole maintained its ability to reduce DOPAC but acquired that of reducing DA. Moreover, raclopride and quinpirole, when locally perfused into the mPFC of rats subjected to noradrenergic denervation, were able to increase and decrease, respectively, extracellular DA levels, while being ineffective in control rats. Transient inactivation of noradrenergic neurons by clonidine infusion into the locus coeruleus, a condition where NET is preserved, was found to reduce extracellular NE and DA in the mPFC, whereas noradrenergic denervation, a condition where NET is eliminated, almost totally depleted extracellular NE but increased DA. Both transient inactivation and denervation of noradrenergic neurons were found to reduce the number of spontaneously active DA neurons and their bursting activity in the VTA. The results indicate that DA released in the mPFC by dopaminergic terminals is not detected by microdialysis unless DA clearance from extracellular space is inactivated. They support the hypothesis that noradrenergic terminals are the main source of DA measured by microdialysis in the mPFC during physiologically relevant activities.

## Introduction

Dopaminergic and noradrenergic projections from the ventral tegmental area (VTA) and the locus coeruleus (LC), respectively, converge to the prefrontal cortex (PFC), where they play a key role in the cognitive and motivational functions of this region (Descarries et al., 1987; Seguela et al., 1990; Phillips et al., 2004; Xing et al., 2016). Accordingly, impairments of catecholamine transmission in the PFC have been implicated in the cognitive and emotional deficits in schizophrenia (Bird et al., 1979; van Kammen and Kelley, 1991; Tassin, 1992; Slifstein and Abi-Dargham, 2017; Rao et al., 2019), depressive disorders (Bunney and Davis, 1965; Schildkraut, 1965; Kapur and Mann, 1992; Dunlop and Nemeroff, 2007; El Mansari et al., 2010), substance use disorder (Koob and Nestler, 1997; Wise, 1998; Volkow et al., 2007; Weinshenker and Schroeder, 2007; Aston-Jones and Kalivas, 2008) and attention deficit/hyperactivity disorder (Bymaster et al., 2002; Engert and Pruesner, 2008; Holzer et al., 2013; Gold et al., 2014).

Conversely, the efficacy of atypical antipsychotics, including clozapine and olanzapine, in ameliorating the negative symptoms and cognitive deficits in schizophrenia has been attributed to their ability to increase norepinephrine (NE) and dopamine (DA) release in the PFC (Meltzer and Stahl, 1976; Moghaddam and Bunney, 1990; Lidow et al., 1998; Melis et al., 1999; Gessa et al., 2000; Devoto et al., 2003).

Different pharmacological treatments, such as psychostimulants (Darracq et al., 1998), antipsychotic drugs (Westerink et al., 1998; Devoto et al., 2003), fluoxetine (Bymaster et al., 2002), stress (Finlay et al., 1995; Feenstra, 2000), aversive and rewarding conditioned and unconditioned stimuli (see Fenstra, 2000) have been shown to produce concomitant and parallel changes in extracellular NE and DA in the PFC, suggesting a cross-talk between dopaminergic and noradrenergic systems to modulate the activity of target neurons in the PFC (Guiard et al., 2008; Masana et al., 2011; Chandler et al., 2014; Xing et al., 2016; Ranjbar-Slamloo and Fazlali, 2020).

A key role has been attributed to the NE transporter (NET) and α_2_-adrenoceptors in governing the cross-talk between dopaminergic and noradrenergic inputs to the PFC. Thus, in view of the low level of DA transporter (Sesack et al., 1998) and abundance of NET in the PFC (Freed et al., 1995; Miner et al., 2003), it has been suggested that DA uptake from extracellular spaces is carried out almost exclusively by NET (Carboni et al., 1990; Pozzi et al., 1994; Gresch et al., 1995; Moron et al., 2002), and that DA competes with NE for the same transporter for which DA has the same affinity as NE (Raiteri et al., 1977; Horn, 1990). Accordingly, an increase of extracellular NE would reduce DA uptake, leading to extracellular DA increase and, vice versa, a reduced NE would facilitate DA clearance leading to a reduction of extracellular DA levels (Carboni et al., 1990).

In addition to the above hypotheses, it has been suggested that presynaptic α_2_-heteroreceptors control catecholamine release from dopaminergic terminals, so that their blockade and stimulation would respectively result in the increase or decrease of DA in the PFC (Pozzi et al., 1994; Gresch et al., 1995; Hertel et al., 1999).

At variance from these hypotheses, early studies from our laboratory suggest that extracellular DA in the cerebral cortex originates other than from dopaminergic also from noradrenergic terminals, where DA would act both as the precursor and co-transmitter of NE (Devoto et al., 2001; Devoto et al., 2002; Devoto et al., 2003; Devoto et al., 2004; Devoto and Flore, 2006).

Accordingly, we have recently demonstrated that central noradrenergic denervation prevented the elevation of extracellular DA in the mPFC elicited by the α_2_-adrenoceptor antagonist atipamezole, suggesting that noradrenergic terminals are the primary source of DA released by α_2_-adrenoceptor antagonists in the mPFC (Devoto et al., 2019).

The present study was aimed to test the hypothesis that DA released by dopaminergic terminals in the mPFC is readily taken up from extracellular fluid by the NET into NE nerve terminals, so that DA cannot be detected by *in vivo* microdialysis unless its clearance from extracellular space is inactivated.

This hypothesis might provide an answer to several questions, such as why D2-receptor antagonists, including haloperidol, raclopride, amisulpride, and sulpiride do not, or only weakly, increase extracellular DA in the prefrontal cortex (Moghaddam and Bunney, 1990; Di Giovanni et al., 1998; Gessa et al., 2000; Devoto et al., 2001; Westerink et al., 2001; Tanda et al., 2015), why the D2-receptor agonist quinpirole does not reduce dialysate levels of DA in the mPFC (Devoto et al., 2001; Devoto et al., 2003) and why noradrenergic denervation fails to reduce extracellular DA in the mPFC (Carboni et al., 1990; Pozzi et al., 1994; Yamamoto and Novotney, 1998; Valentini et al., 2004; Devoto et al., 2015; Devoto et al., 2019), which should be otherwise expected according to the hypothesis of the noradrenergic source of extracellular DA in the mPFC.

These questions are relevant not so much to the technical limitations of microdialysis as to the interpretation of DA measurements by *in vivo* microdialysis, in the sense that DA changes attributed to meso-prefrontal dopaminergic transmission may be indeed mediated by noradrenergic one.

## Materials and Methods

### Subjects

Male Sprague-Dawley rats (ENVIGO, Italy) weighing 250–350 g, were group-housed and kept on a regular 12:12 h light/dark cycle, in temperature- and humidity-controlled facilities, with food and water available ad libitum. The experimental protocols were conducted to minimize pain and suffering and to reduce the number of animals used. Experiments were approved by the Animal Ethics Committees of the University of Cagliari and were carried out in accordance with the European Directive on the protection of animals used for scientific purposes (2010/63/EU). In total 126 animals were used.

### Noradrenergic Denervation

Central noradrenergic system ablation was achieved as previously described (Devoto et al., 2015; Devoto et al., 2019) by the administration of the selective neurotoxin anti-dopamine-β-hydroxylase saporin (aDBH). Briefly, rats were anesthetized with Equithesin (0.97 g pentobarbital, 2.1 g MgSO_4_, 4.25 g chloral hydrate, 42.8 ml propylene glycol, 11.5 ml 90% ethanol in 100 ml; 5 ml/kg, IP) and placed in a Kopf stereotaxic apparatus. A hole was drilled in the skull, directed to the lateral ventricle [AP −1.0, L ± 1.5 from the bregma, V −4.3 from skull, coordinates according to Paxinos and Watson (2007)] for administration of 5 µg/5 µl immunotoxin (n = 39, aDBH rats) or 5 µl vehicle (n = 40, control rats), with a 10 µl syringe operated by a CMA/100 microinjection pump (CMA Microdialysis, Stockholm, Sweden) at 1 µl/min during 5 min, followed by 2 min pause before slowly withdrawing the needle. Injections were randomly distributed into either the left or right lateral ventricles. Rats were given antibiotic therapy (enrofloxacin, Bayer HealthCare, Shawnee Mission, KS) for five days and allowed to recover in their home cages for fifteen to eighteen days before the experiment. Some electrophysiology or microdialysis experiments were performed in intact rats (n = 35).

### Microdialysis

Rats were stereotaxically implanted with vertical microdialysis probes (membrane AN 69-HF, Hospal-Dasco, Bologna, Italy; cut-off 40,000 Daltons, 4 mm active membrane length), in the mPFC (AP +3.0, L ± 0.6, V −6.5 from the bregma, according to the coordinates of Paxinos and Watson, 1997), under Equithesin anesthesia. The day after probe implantation, artificial cerebrospinal fluid (aCSF: 147 mM NaCl, 4 mM KCl, 1.5 mM CaCl_2_, 1 mM MgCl_2_, pH 6–6.5) was pumped through the dialysis probes at a constant rate of 1.1 µl/min *via* a CMA/100 microinjection pump (Carnegie Medicine, Stockholm, Sweden) in freely moving animals, and dialysate samples were collected every 20 min. A subgroup of 11 rats was perfused with aCSF containing 10 µM nisoxetine. Drugs were administered after stable extracellular levels were obtained, i.e. three consecutive samples with a variance not exceeding 15%. The average of these three values was considered as baseline and used as 100% for the subsequent calculation of the variations induced by the administration of drugs. NE, DOPAC and DA were simultaneously analyzed by HPLC with electrochemical detection, by HPLC systems equipped with 3.0 × 150 mm C18 (3.5 µ) Symmetry columns (Waters, Milan, Italy), maintained at 40°C by Series 1100 thermostats (Agilent Technologies, Waldbronn, Germany), and ESA Coulochem II detectors (Chelmford, MA, USA). The mobile phase consisted of 80 mM Na_2_HPO_4_, 0.27 mM EDTA, 0.6 mM sodium octyl sulfate, 7% methanol, 4% acetonitrile, pH 2.4 with H_3_PO_4_, delivered at 0.3 ml/min; the Coulochem analytical cell first electrode was set at +200 mV, the second at −200 mV. Quantification was performed by recording the second electrode signal. Under these conditions, NE and DA detection limits (signal to noise ratio 3:1) were 0.3 pg per injection on column. In most aDBH-lesioned rats, NE concentration fell below the HPLC detection limit, therefore the actual NE value was in the range of 0 to 0.3 pg. To avoid bias in data analysis, according to the suggestion by McKittrick and Abercrombie (2007) the samples whose NE was not detectable were attributed the theoretical value of 0.15 pg, chosen taking into account that the actual value may range between the two extremes.

A sub-group of animals was implanted with a microdialysis probe into the locus coeruleus (LC, AP 11.1 mm, L ± 1.2 mm, V −8.2 mm from cortical surface, with an inclination of 16°, 2 mm dialyzing membrane), homolaterally to mPFC or VTA to be analyzed.

On completion of testing, rats were sacrificed by Equithesin overdose, the brains removed and sectioned by a cryostat (Leica CM3050 S) in 40 µm thick coronal slices to verify locations of dialysis probes. No animal was found with errant location of the device.

### Tissue Catecholamine and DOPAC Evaluation

To measure tissue contents of NE, DA and DOPAC, rats were sacrificed by decapitation 15 days after aDBH (n = 6) or vehicle (n = 6) i.c.v. injection; brains were rapidly removed and placed on a brain cutting block maintained on ice. The mPFC was dissected out from 2 mm slices, immediately frozen on dry ice, and stored at –80°C until processing for catecholamine content. Briefly, tissues were weighed, homogenized by sonication in 0.1 M HClO_4_ (1:20 weight tissue per solvent volume), centrifuged at 10,000*g*, the supernatant filtered using microspin centrifuge tubes (0.22 µm nylon filter), and directly injected into the HPLC in the same analytical conditions described for microdialysis experiments. Data were expressed as pg per mg tissue.

### *In Vivo* Single-Unit Recordings

Rats were anaesthetized with urethane 1.3 g/kg, IP. Rats were placed in a stereotaxic apparatus (Kopf, Tujunga, CA, USA) with their body temperature maintained at 37 ± 1°C by a heating pad. The recording electrode was placed above the VTA (5.1–5.7 posterior to bregma, 0.2–0.6 mm lateral to midline, 7.0–8.0 mm from cortical surface), according to the stereotaxic rat brain atlas of Paxinos and Watson (Paxinos and Watson, 2007). Single unit activity of neurons was recorded extracellularly (bandpass filter 0.1–10,000 Hz) with glass micropipettes filled with 2% Pontamine sky blue dissolved in 0.5 M sodium acetate. Individual action potentials were isolated and amplified by means of a window discriminator (Neurolog System, Digitimer, Hertfordshire, UK) and displayed on a digital storage oscilloscope (TDS 3012, Tektronics, Marlow, UK). Experiments were sampled online and offline with Spike2 software by a computer connected to CED1401 interface (Cambridge Electronic Design, Cambridge, UK). Dopamine neurons were isolated and identified according to previously described electrophysiological characteristics (Grace and Bunney, 1983; Ungless et al., 2004). VTA dopamine neurons were recorded only when criteria for identification were fulfilled (firing rate ≤ 10 Hz, duration of action potential ≥ 2.5 ms). Bursts were defined as the occurrence of two spikes at interspike interval < 80 ms and terminated when the interspike interval exceeded 160 ms.

To analyze cell population activity, the electrode was passed within each area in 12 predetermined tracks separated by 200 µm (5.1 to 5.7 mm posterior to bregma; 0.2 to 0.6 mm lateral to midline) and the total number of active cells encountered in each area was divided by the number of tracks.

### Drugs and Treatments

The immunotoxin aDBH was purchased from Advanced Targeting System (San Diego, CA, USA); nisoxetine hydrochloride and clonidine hydrochloride were from Tocris (Bristol, UK), quinpirole hydrochloride and raclopride tartrate were from Sigma-Aldrich. 2. A single, full effective dose of each drug was chosen, according to literature and our previous experience, to describe a qualitative rather than a quantitative effect (Nisoxetine: Chen and Reith, 1994; Rowley et al., 2000; Devoto et al., 2019. Quinpirole: Devoto et al., 2001; Marinelli et al., 2006. Clonidine: Devoto et al., 2001; Gobbi et al., 2001. Raclopride: Wong et al., 2018; Devoto et al., 2019). Drugs were dissolved in sterile distilled water, and IP or subcutaneously (SC) administered in a volume of 1 ml/kg body weight. Sub-groups of animals were treated by infusion through microdialysis probe with 20 µM clonidine in aCSF into the LC, or with 10 µM nisoxetine in aCSF into the mPFC.

### Statistical Analysis

Data were analyzed by unpaired Student’s t test, with or without Welch’s correction, one-way or two-way repeated measures ANOVA, as appropriate and specified in Results. Post-hoc multiple comparisons were made using Sidak’s multiple comparison test. In all cases, P < 0.05 was considered significant. Statistic was performed using GraphPad Prism version 7.00 (GraphPad Software, La Jolla California USA, www.graphpad.com).

## Results

### Raclopride Increases Extracellular DA in the mPFC After Loss or Inactivation of NET

**Figure 1:**
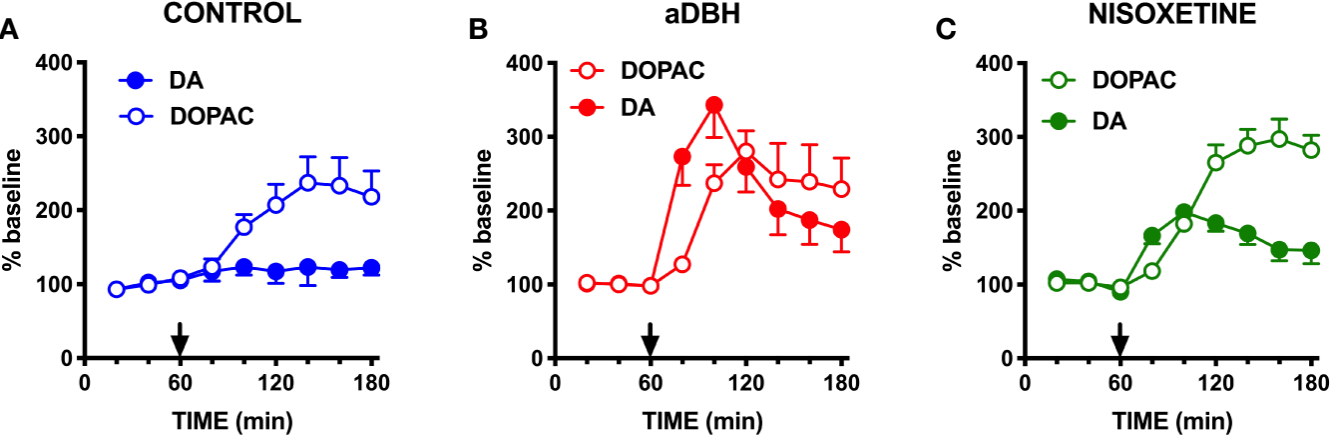
Effect of raclopride on extracellular DA and DOPAC levels in **(A)** control rats, **(B)** in aDBH denervated rats and **(C)** during continuous nisoxetine perfusion into the mPFC. Raclopride was administered at time point 60 min, as indicated by the arrow. Data are expressed as percent of baseline and are the mean ± SEM obtained from 4 control rats **(A)**, 6 aDBH-lesioned rats **(B)** and 5 nisoxetine-perfused rats **(C)**. One-way repeated measures ANOVA indicated a significant effect of raclopride on DOPAC (F_(1.223, 3.668)_ = 12.54, P = 0.0262) but not DA (F_(1.366, 4.097)_ = 0.9247, P = 0.4255) in control rats. In aDBH lesioned rats one-way repeated measures ANOVA indicated a significant effect of raclopride on for both DOPAC (F_(1.306, 6.532)_ = 10.48, P = 0.0128) and DA (F_(1.654, 8.268)_ = 18.06, P = 0.0013). In nisoxetine perfused rats, one-way repeated measures ANOVA indicated a significant effect of raclopride on DOPAC (F_(2.379, 9.515)_ = 43.01, P < 0.0001) and DA (F_(1.905, 7.62)_ = 18.12, P = 0.0014). Data were further analyzed by two-way repeated measures ANOVA, which indicated a significant effect in time x experimental condition interaction (F_(10, 60)_ = 8.08, P < 0.0001), time (F_(2.65, 31.8)_ = 15.7, P < 0.0001) and experimental condition (F_(2, 12)_ = 5.55, P = 0.0197) for DA and in time × experimental condition interaction (F_(10, 60)_ = 2.13, P = 0.0359), time (F_(1.64, 19.7)_ = 24.2, P < 0.0001) but not in experimental condition (F_(2, 12)_ = 0.508, P = 0.6143) for DOPAC.

**Figure 2:**
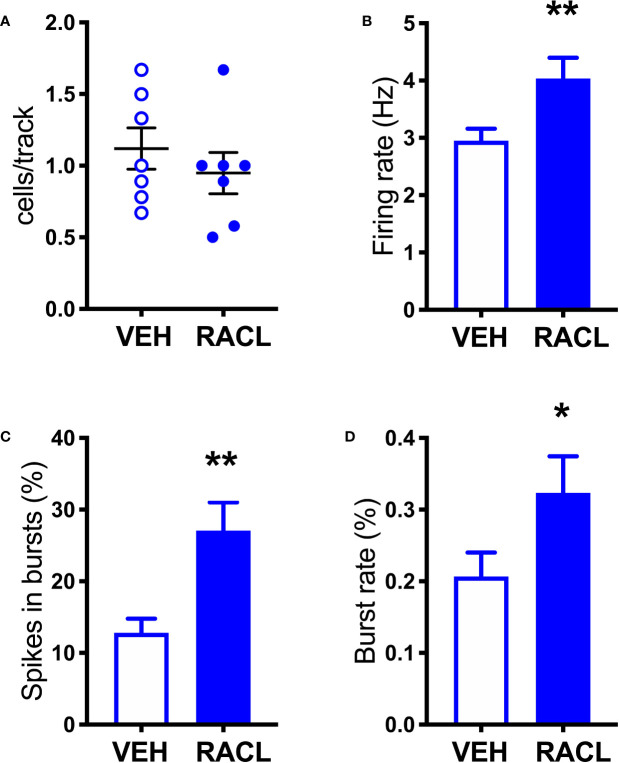
Raclopride increases the electrical activity of DA cells in control rats. **(A)** Raclopride did not change the number of spontaneously active VTA DA neurons per track (Veh 1.1 ± 0.1, n = 7; Racl: 0.9 ± 0.1, n = 7, t = 0.8410, df = 12, P = 0.41). **(B)** Raclopride increased DA neuron firing rate (Veh: 2.9 ± 0.2 Hz, n = 61, Racl:4.0 ± 0.4 Hz, n = 31, t = 2.795, df = 90, P = 0.006); **(C)** the percentage of spikes in bursts (Veh: 12.8 ± 2.0%, n = 61, Racl: 27.1 ± 3.9, n = 31, t = 3.624, df = 90, P = 0.0005), **(D)** burst rate (Veh: 0.2 ± 0.03, n = 48, Racl: 0.3 ± 0.05, n = 29, t = 2.002, df = 75, P = 0.048). *,P < 0.05, **P < 0.01 (unpaired Student’s t-test with or without Welch’s correction, as appropriate).

As shown in **Figure 1**, in control rats raclopride (0.5 mg/kg, IP) increased extracellular DOPAC by about 140% in the mPFC (**Figure 1A**) and activated the firing rate and the bursting activity (measured ad spikes in burst and burst rate) of DA neurons in the VTA (**Figure 2**). However, consistent with previous results with different D2-receptor antagonists (Moghaddam and Bunney, 1990; Kuroki et al., 1999; Gessa et al., 2000; Devoto et al., 2001; Tanda et al., 2015), raclopride failed to modify extracellular DA in the mPFC (**Figure 1A**).

To verify whether DA uptake by NET into noradrenergic terminals prevented DA from being detected by microdialysis, raclopride was administered in rats whose NET was either eliminated by noradrenergic denervation with the neurotoxin aDBH or inactivated by nisoxetine.

**Figure 3:**
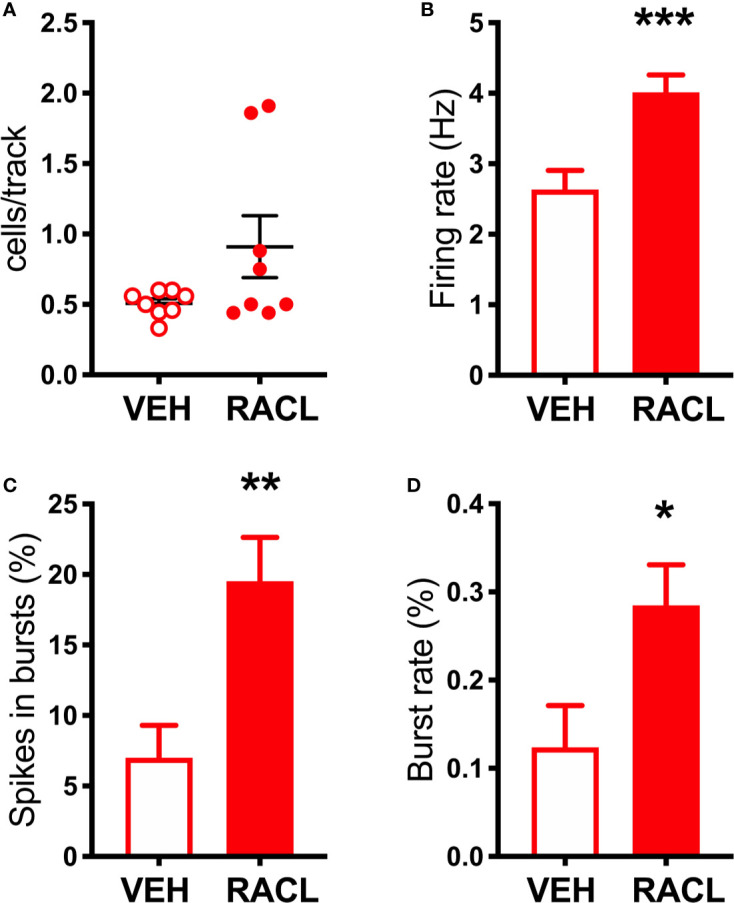
Raclopride increases the electrical activity of DA cells in aDBH-lesioned rats **(A)** Raclopride did not significantly affect the number of spontaneously active VTA DA neurons per track (Veh 0.5 ± 0.03, n = 8; Racl: 0.9 ± 0.2, n = 8, t = 1.812, df = 14, P = 0.09). **(B)** Raclopride increased DA neuron firing rates (Veh: 2.6 ± 0.3 Hz, n = 32, Racl:4.0 ± 0.2 Hz, n = 59, t = 3.558, df = 89, P = 0.0006); **(C)** the percentage of spikes in bursts (Veh: 7.0 ± 2.3%, n = 32, Racl: 19.5 ± 3.0, n = 59, t = 2.765, df = 89, P = 0.0069), **(D)** burst rate (Veh: 0.12 ± 0.04, n = 20, Racl: 0.28 ± 0.04, n = 49, t = 2.057, df = 67, P = 0.046). *P < 0.05, **P < 0.01, ***P < 0.001 (unpaired Student’s t-test with or without Welch’s correction, as appropriate).

As shown in **Figure 1B**, in denervated rats raclopride increased extracellular DOPAC (by 170%) in the mPFC and, as in control rats, activated the firing rate and bursting activity (measured ad spikes in burst and burst rate) of DA neurons in the VTA (**Figure 3**) but, unlike in control rats, raclopride also increased extracellular DA in the mPFC by 230% (**Figure 1B**).

As **Table 1** shows, consistent with earlier results (Devoto et al., 2015; Devoto et al., 2019), the ICV injection of aDBH (0.5 µg/5µl) depleted tissue and extracellular NE almost totally and did not modify tissue DA and DOPAC. Notably, in apparent contrast to our hypothesis that noradrenergic terminals supply DA in the mPFC, noradrenergic denervation did not reduce extracellular DA (actually increased it by about 38%) and reduced extracellular DOPAC by 50%.

**Table 1:** Tissue and extracellular catecholamine levels in the medial prefrontal cortex of rats treated with aDBH or its vehicle.

Treatment	Tissue level (pg/mg T)			Extracellular level (pg/sample)		
	NE	DA	DOPAC	NE	DA	DOPAC
Control	262.9 ± 35.5	42.3 ± 9.5	30.5 ± 4.0	2.9 ± 0.3	1.9 ± 0.2	235.0 ± 32.2
aDBH	ND	47.4 ± 8.6	28.0 ± 2.9	0.3 ± 0.1***	2.6 ± 0.2*	118.6 ± 25.2**

Tissue values are expressed as pg/mg fresh weight and are the means ± SEM of 6 rats. Dialysate levels are means ± SEM of values obtained from 26 control and 23 aDBH-treated rats. For each rat, mean extracellular catecholamine and DOPAC values were calculated from three consecutive samples not differing more than 15%, and are expressed as pg per 20 µl sample. NE values were lower than detection limit in 6 out of 6 tissue samples and in 16 out of 21 extracellular basal levels. The latter were conventionally calculated as 0.15 pg, as detailed in Methods. Unpaired t test with Welch’s correction evidenced significant differences between control and lesioned animal in tissue NE (t = 10.04 df = 7.003, P < 0.0001), but not tissue DA (t = 0.3966, df = 10, P = 0.700) and DOPAC (t = 0.4933 df = 10, P = 0.6325), while extracellular values were significantly different for NE (t = 8.0 df = 22.37, P < 0.0001), DA (t = 2.132, df = 47, P = 0.0383) and DOPAC (t = 2.474, df = 45, P = 0.0086).

*P < 0.01; **P < 0.01; ***P < 0.0001 vs control rats (2-tailed unpaired t test).

ND, not detectable.

As shown in **Figure 1C**, raclopride (0.5 mg/kg, IP) administration to rats whose NET was inactivated by nisoxetine (10 µM, locally infused into the mPFC by reverse dialysis), increased both extracellular DOPAC and DA to approximately 200% and 300% above the levels produced by nisoxetine alone (**Figure 1C**).

As expected from previous results (Carboni et al., 1990; Linnér et al., 2001; Bymaster et al., 2002), NET inhibition by nisoxetine increased extracellular NE and DA by about 600% and 170%, respectively, and did not increase, but actually reduced, extracellular DOPAC in the mPFC (**Table 2**).

**Table 2:** Extracellular catecholamine levels in the medial prefrontal cortex of rats locally perfused with nisoxetine (10 µM) and control rats.

Treatment	Extracellular level (pg/sample)		
	NE	DA	DOPAC
CONTR	2.19 ± 0.8	1.96 ± 0.4	304.1 ± 63.2
NIS	15.4 ± 1.1****	5.36 ± 0.4****	231.5 ± 30.1

Dialysate levels are means ± SEM of values obtained in 9 control and 11 nisoxetine-perfused rats. For each rat, mean extracellular catecholamine and DOPAC values were calculated from three consecutive samples with a variance not exceeding 15%, and are expressed as pg per 20-µl sample. Values are expressed as pg per sample. Unpaired t test with Welch’s correction evidenced a significant difference between control and nisoxetine-perfused rats in extracellular NE (t = 9.704, df = 12.64, P < 0.0001) and DA (t = 5.861 df = 17.94, P < 0.0001), but not DOPAC (t = 1.102, df = 18, P = 0.285).

****P < 0.0001 vs control rats (2-tailed unpaired t test with Welch’s correction).

### The D2-Receptor Agonist Quinpirole Decreases Extracellular DA in the mPFC After Loss or Inactivation of NET

**Figure 4:**
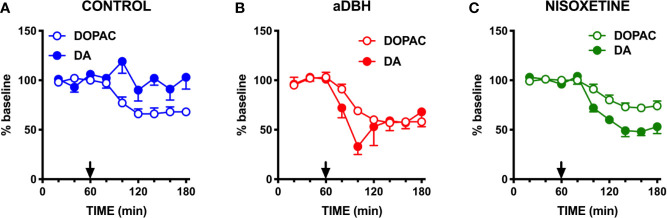
Effect of quinpirole on extracellular DA and DOPAC levels in **(A)** control rats, **(B)** in aDBH denervated rats and **(C)** during continuous nisoxetine perfusion into the mPFC. Quinpirole was administered at time point 60 min, as indicated by the arrow. Data are expressed as percent of mean basal value and are the mean ± SEM obtained from 5 control, 3 (aDBH) and 6 nisoxetine rats. One-way repeated measures ANOVA indicated a significant effect of quinpirole on DOPAC (F_(2.14, 8.56)_ = 24.52, P = 0.0003) but not DA (F_(2.538, 10.15)_ = 1.329, P=0.3143) in control rats; for DA (F_(1.958, 9.788)_ = 28.88, P < 0.0001) and DOPAC (F_(1.917, 9.587)_ = 14.87, P = 0.0012) in nisoxetine-perfused rats and for both DA (F_(1.815, 3.631)_ = 8.861, P = 0.0409) and DOPAC (F_(1.245, 2.49)_ = 28.13, P = 0.0202) in a-DBH-lesioned rats. Data were further analyzed by two-way repeated measures ANOVA, which indicated a significant effect in time x experimental condition interaction (F_(10, 55)_ = 4.54, P = 0.0001), time (F_(2.14, 23.5)_ = 5.52, P = 0.0097) and experimental condition (F_(2, 11)_ = 14.0, P = 0.001) for DA, and in time (F_(1.88, 20.6)_ = 37.7, P = 0.0097) and experimental condition (F_(2, 11)_ = 4.66, P = 0.0342, but not in time x experimental condition interaction (F_(10, 60)_ = 0.752, P = 0.6732) for DOPAC.

**Figure 5:**
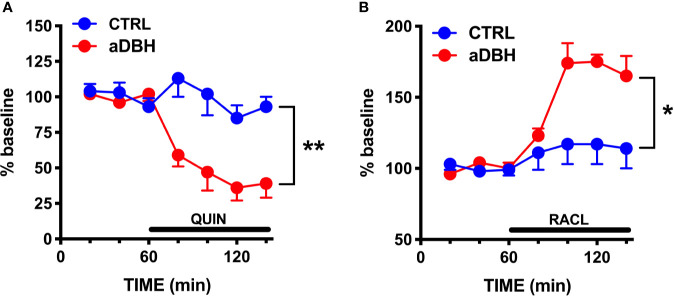
Effect of quinpirole **(A)** and raclopride **(B)** infusion into the mPFC on extracellular DA levels of control (CTRL) and aDBH-lesioned (aDBH) rats. Data are expressed as % of baseline and are the mean ± SEM of samples collected from 5 (aDBH) and 4 (CTRL) rats for quinpirole (10 µM) and from 5 (aDBH) and 6 (CTRL) rats for raclopride (100 µM). Drugs were perfused into the mPFC from time point 60 to the end of experiment (horizontal bar). Two-way ANOVA with repeated measures indicated a significant difference in experimental condition (F_(1,7)_ = 22.4; P = 0.0021) but not in time (F_(3, 21)_ = 2.56, P = 0.0824) and time x experimental condition interaction (F_(3, 21)_ = 0.697, P = 0.5645) for quinpirole effect, and in experimental condition (F_(1,9)_ = 9.03, P = 0.0148), time (F_(3,27)_ = 5.93, P = 0.0030) and time x experimental condition interaction (F_(3,27)_ = 3.69, P = 0.0240) for raclopride effect. **P = 0.0148; *P = 0.0021.

Consistent with previous results (Devoto et al., 2001; Devoto et al., 2002; Devoto et al., 2003), quinpirole administration (0.1 mg/kg, SC) to control rats reduced extracellular DOPAC by 30% but failed to modify extracellular DA in the mPFC (**Figure 4A**), even though this dose is able to fully inhibit the activity of both fast- and slow-firing VTA dopaminergic neurons (Marinelli et al., 2006; Perez and Lodge, 2012). To clarify whether quinpirole-induced changes in extracellular DA remained undetected because of DA being taken-up by NET, the effect of quinpirole (0.1 mg/kg, SC) was tested in rats whose NET was eliminated by noradrenergic denervation or inactivated by nisoxetine. As shown in **Figure 4B**, after noradrenergic denervation quinpirole reduced extracellular DOPAC (by 40%), like in control rats, but, unlike the latter, also reduced extracellular DA by 70%. In rats whose NET was inactivated by nisoxetine (10 µM, locally infused into the mPFC by reverse dialysis), quinpirole not only reduced extracellular DOPAC (by 30%), like in control rats, but did also reduce extracellular DA by 50% in the mPFC (**Figure 4C**). Notably, quinpirole and raclopride perfused *via* reverse microdialysis into the mPFC reproduced the same effects seen after systemic administration, in that quinpirole (10 µM) and raclopride (100 µM) failed to modify extracellular DA in control rats but quinpirole reduced by 60% and raclopride increased by 75% extracellular DA after noradrenergic denervation (**Figure 5**). These results are consistent with the notion that D2-receptor on presynaptic terminals play a key role in the action of D2-receptor agonists and antagonists (Anzalone et al., 2012; Ford, 2014).

### Transient Inactivation, Unlike Denervation, of Noradrenergic Neurons Reduces Extracellular DA in the mPFC

To clarify whether the loss of NET prevented noradrenergic denervation from reducing extracellular DA in the mPFC, this condition was compared with the transient inactivation of noradrenergic neurons, produced by clonidine infusion into the locus coeruleus, a condition in which NET activity is preserved.

**Figure 6:**
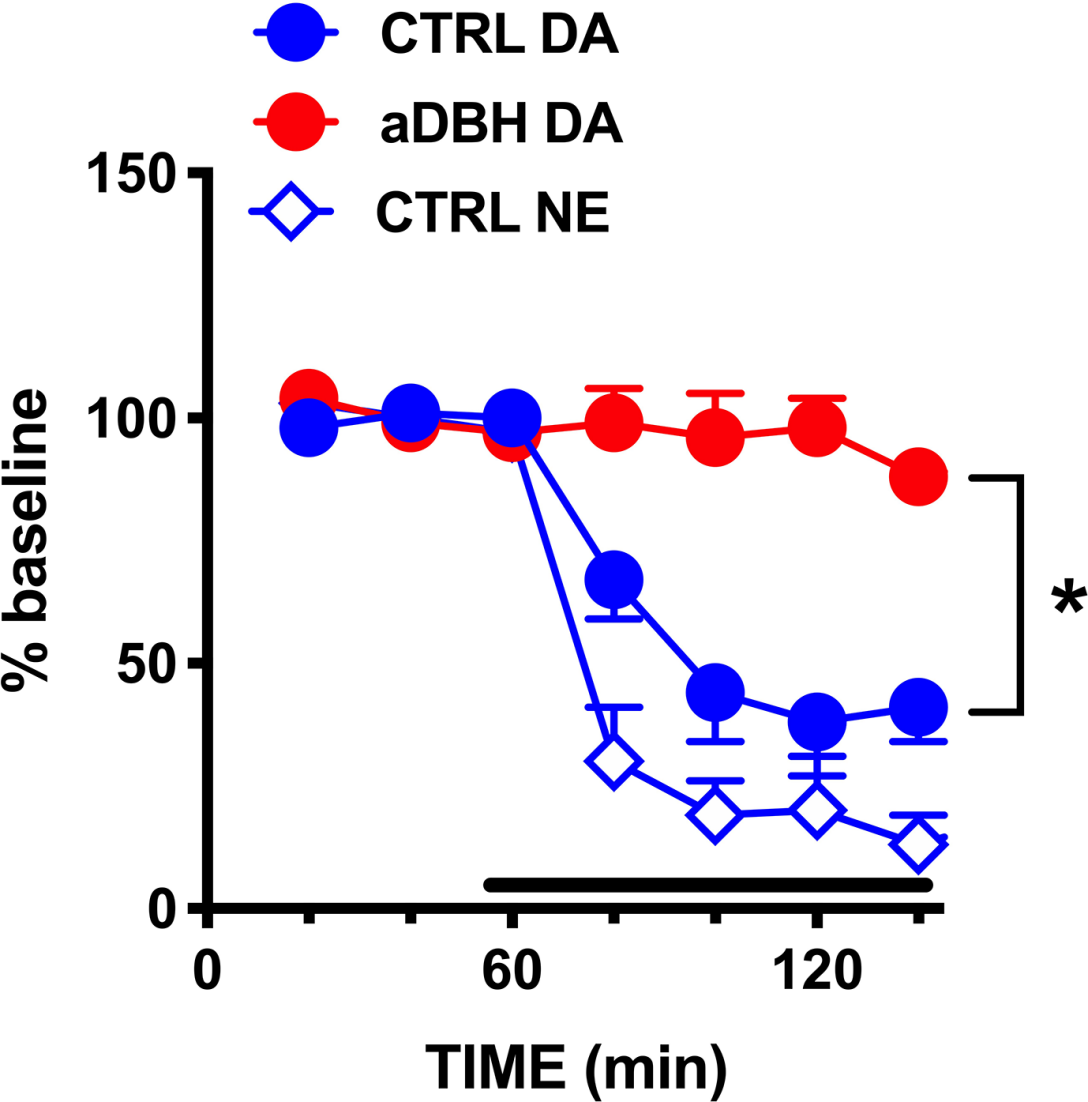
Effect of clonidine infusion into the LC on extracellular DA and NE in the mPFC. Data are expressed as % of baseline and are the mean ± SEM of samples collected from 4 (aDBH) and 7 (CTRL) rats. Clonidine was perfused into the LC homolateral to the mPFC from time point 60 to the end of experiment (horizontal bar). Two-way ANOVA with repeated measures of DA levels indicated a significant effect of aDBH treatment (F_(1,9)_ = 30.5; P = 0.0004), but not in time (F_(2, 18)_ = 1.93, P = 1737, and in time x experimental condition interaction (F_(3, 27)_ = 0.989, P = 0.4126). *P = 0.0004

As shown in **Figure 6**, clonidine (20 µM) reduced extracellular NE and DA by 80% and 60%, respectively, in the mPFC of intact rats, but did not modify extracellular DA in aDBH-lesioned rats.

### Transient Inactivation and Denervation of Noradrenergic Neurons Similarly Reduce the Firing of DA Neurons

**Figure 7:**
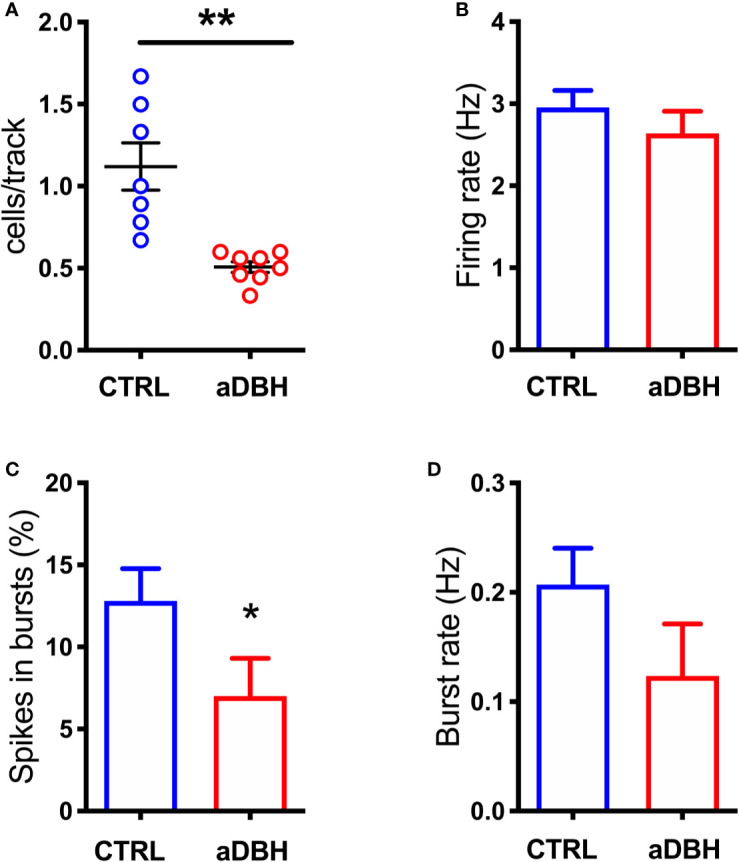
Effect of aDBH-induced noradrenergic denervation on firing of VTA DA neurons. The scatter plot **(A)** shows the number of spontaneously active VTA DA neurons encountered by the electrode per each track (unpaired Student’s t-test with Welch correction, t = 4.138, df = 6.609, P= 0.0025). Graph histograms represent **(B)** individual DA neuron firing rates, in CTRL and aDBH-treated rats (control group: 2.954 ± 0.207 Hz, n = 61; aDBH-treated group: 2.637 ± 0.27 Hz, n = 32; t = 0.913 df = 91, P = 0.364); **(C)** percentage of spikes in bursts (control group: 12.8 ± 1.974%, n = 61; aDBH-treated group: 7.01 ± 2.29%, n = 32; t = 1.814 df = 91, P = 0.0365) and **(D)** burst rate (control group: 0.207 ± 0.033 Hz, n = 48; aDBH-treated group: 0.124 ± 0.048 Hz, n = 20, t = 1.391 df = 66, P = 0.169). Data are expressed as mean ± SEM and were analyzed with unpaired Student’s t-test. *P < 0.05; **P < 0.01.

**Figure 8:**
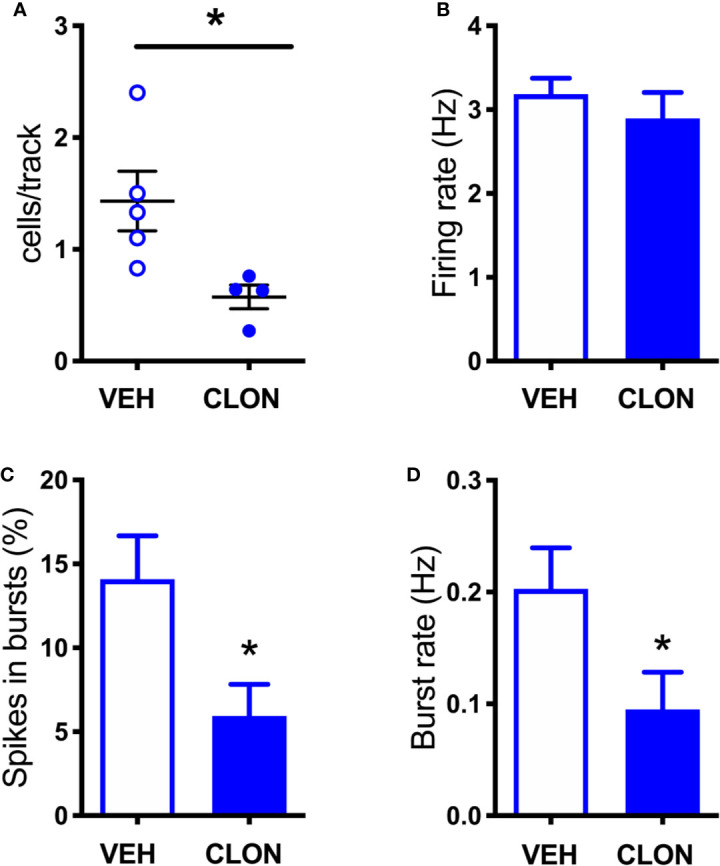
Effect of clonidine continuously infused into the LC on the firing of VTA DA neurons. **(A)** number of spontaneously active VTA DA neurons per track in vehicle (VEH) and clonidine -treated rats (t = 2.707 df = 7, P = 0.0303). **(B)** individual DA neuron firing rates (control group: 3.185 ± 0.19 Hz, n = 58; clonidine-treated group: 2.896 ± 0.308 Hz, n = 25; t = 0.816, df = 81, P = 0.4168). **(C)** percentage of spikes in bursts (control group: 14.08 ± 2.59%, n = 47; clonidine-treated group: 5.94 ± 1.88%, n = 25; t = 2.546, df = 80.06, P = 0.0128). **(D)** burst rate (control group: 0.203 ± 0.037 Hz, N = 47; clonidine-treated group: 0.095 ± 0.033 Hz, n = 21; t = 2.175 df = 59.89, P = 0.0336). *P < 0.05 (unpaired Student’s t-test with or without Welch’s correction, as appropriate).

To rule out the possibility that differences in DA release in the mPFC between denervation and transient inhibition might depend on differences in the activity of dopaminergic neurons, the electrical activity of DA neurons in the VTA in the two conditions was tested. As shown in **Figures 7**, **8**, denervation and transient inactivation of noradrenergic neurons produced the same changes in the activity of DA neurons, both reducing the number of spontaneously active DA neurons and the bursting activity (assessed as the percentage of spikes in burst and burst rate) but both failed to modify the firing rate of DA neurons.

## Discussion

The present results support the hypothesis that DA released by dopaminergic terminals in the mPFC is readily taken up from extracellular space into noradrenergic terminals by NET, so that it cannot be detected by *in vivo* microdialysis unless its clearance from extracellular space is prevented.

Accordingly, as previously reported for other selective D2-antagonists, including sulpiride, haloperidol, amisulpride, (Moghaddam and Bunney, 1990; Kuroki et al., 1999; Gessa et al., 2000; Devoto et al., 2001; Tanda et al., 2015), raclopride failed to increase extracellular DA in the mPFC, even though it stimulated the electrical activity of DA neurons in the VTA and increased extracellular DOPAC, the deaminated DA metabolite, in the mPFC. However, after NET was inactivated by nisoxetine or eliminated by noradrenergic denervation, raclopride uncovered the ability to increase extracellular DA in the mPFC, as measured by *in vivo* microdialysis, while maintaining that of stimulating the firing of DA neurons in the VTA and increasing extracellular DOPAC in the mPFC.

Importantly, the loss of NET provides a likely explanation for why noradrenergic denervation failed to reduce extracellular DA in the mPFC (Harik, 1984; Carboni et al., 1990; Pozzi et al., 1994; Valentini et al., 2004; Masana et al., 2011; Devoto et al., 2015; Devoto et al., 2019), in apparent contradiction with our hypothesis that noradrenergic terminals supply extracellular DA in the mPFC. Indeed, in accord with this hypothesis, transient inactivation of noradrenergic neurons by clonidine perfusion into the LC, a condition in which NET activity is preserved, produced a profound reduction of extracellular NE and DA in the mPFC. Moreover, consistent with the hypothesis that cortical DA measured by microdialysis originates from noradrenergic terminals, the D2 receptor agonist quinpirole, both given systemically or locally perfused into the mPFC, failed to modify extracellular DA in the mPFC in control rats but, opposite to raclopride, reduced extracellular DA after NET was inactivated by nisoxetine or eliminated by noradrenergic denervation, in line with the hypothesis that extracellular DA from dopaminergic terminals may be detected when DA clearance is impaired.

Notably, as expected from the hypothesis that extracellular DA in the mPFC derives from noradrenergic terminals, in a previous study (Devoto et al., 2019) the α_2_-adrenoceptor agonist clonidine was found to reduce extracellular DA in the mPFC of control rats but to be ineffective after noradrenergic denervation with aDBH.

Our results are consistent with previous observations using *in vivo* microdialysis that quinpirole, locally perfused into the mPFC, lowered extracellular DA in the mPFC, after nomifensine was included in the perfusate (Santiago et al., 1993), and with results in *in vitro* slices of PFC from rats and rabbits showing that different D2-receptor agonists reduced potassium- and electrically stimulated DA release, provided that NET and/or DAT was inhibited by nisoxetine or desmethyl-imipramine (Talmaciu et al., 1986; Wolf and Roth, 1987; Hoffman, 1988).

Our results are not in contrast with the finding that two D3-receptor agonists, (+)-7-OH-DPAT and PD128,907, as well as apomorphine (Ozaki et al., 1989), reduced extracellular DA in the mPFC or frontal cortex (Gobert et al., 1996; Westerink et al., 1998), since the effect of the D3 agonists on DA release in the PFC does not seem to be mediated by D2-autoreceptors (Gobert et al., 1995), while apomorphine is a D2 and D1 receptor agonist (Chipkin et al., 1987) and D1 receptor agonists may reduce extracellular DA in the mPFC (Santiago et al., 1993).

Importantly, results obtained by *in vivo* microdialysis may not reproduce results obtained with voltammetry or chronoamperometry, because microdialysis can measure changes of extracellular DA level with a time resolution of minutes to hours, related to tonic firing of DA neurons, while voltammetry and chronoamperometry can monitor DA released by phasic activation of DA neurons and also single-spike firing with a sub-second time resolution from a closer proximity to the synaptic site of release, likely before DA is cleared by NET (Gobert et al., 1995; Hauber, 2010).

These considerations may explain why, when using chronoamperometry, quinpirole was found to lower extracellular DA in the mPFC (Weber et al., 2018), unlike what we saw by microdialysis.

It is important to note that the hypothesis that DA released by dopaminergic terminals in the mPFC may not be detectable by microdialysis does not contrast the notion that DA is tonically and phasically released by dopaminergic terminals in the mPFC (see Hauber, 2010), and that noradrenergic and dopaminergic systems interact reciprocally both at the level of the LC and VTA and at nerve terminal level in the mPFC (Grenhoff et al., 1993; Guiard et al., 2008; Hauber, 2010; Mejias-Aponte, 2016; Xing et al., 2016; Park et al., 2017; Kielbinski et al., 2019). Indeed, the finding that both transient inactivation and denervation of noradrenergic neurons reduced the activity of DA neurons in the VTA is consistent with the view that NE exerts a tonic excitatory effect on VTA DA cells *via* α_1_-adrenoceptors (Grenhoff et al., 1993; Grenhoff et al., 1993; Park et al., 2017; Kielbinski et al., 2019).

However, the findings that denervation and transient inactivation of noradrenergic neurons similarly reduced the electrical activity of DA neurons in the VTA, but differently modified extracellular DA in the mPFC, while raclopride similarly activated VTA DA neurons in control as in noradrenergic denervated rats, but produced distinct effects on extracellular DA in the mPFC, indicate that changes in extracellular DA, as measured by microdialysis in the mPFC may be dissociated from the electrical activity of DA neurons in the VTA.

Importantly, previous observations (Gessa et al., 2000) indicate that such dissociation may apply to meso-prefrontal DA neurons as well, since haloperidol, clozapine and olanzapine, in doses equally effective in stimulating the electrical activity of meso-prefrontal DA neurons, were found differ in modifying extracellular DA in the mPFC: namely, D2-receptor antagonist haloperidol failed to modify extracellular DA in the mPFC, while clozapine and olanzapine, that inhibit both D2- and α_2_-receptors (Baldessarini et al., 1992; Bymaster et al., 1996; Devoto et al., 2003; Tauscher et al., 2004), increased extracellular DA, supposedly from dopaminergic and noradrenergic terminals (Gessa et al., 2000).

An important corollary of our results regards the formation and function of extracellular DOPAC in the mPFC. The finding that extracellular DOPAC paralleled the changes of dopaminergic activity supports the view that extracellular DOPAC, unlike DA, is a reliable indicator of DA activity in the mPFC.

Extracellular DOPAC in the mPFC is thought to originate from the oxidation by MAO-A of DA captured by DAT and NET into dopaminergic and noradrenergic terminals, respectively (Mazei et al., 2002; Kaenmaki et al., 2010; Tammimaki et al., 2016).

The finding that extracellular DOPAC was reduced by 50% after noradrenergic denervation and was slightly, although not significantly, reduced after NET inhibition by nisoxetine, in spite of the marked increase of extracellular DA, indicates that noradrenergic terminals substantially contribute to DOPAC formation in the mPFC.

As to the source of DA to form DOPAC by noradrenergic terminals, previous results (Devoto et al., 2003) indicated that DOPAC measured by microdialysis in the mPFC was five times higher than that in the occipital cortex, a region equally innervated by noradrenergic afferences but with scarce dopaminergic terminals. These results suggest that the majority of DA oxidized to DOPAC by noradrenergic terminals originates from dopaminergic terminals.

Our results suggest a mechanism by which atypical antipsychotics rescue the impaired dopaminergic functioning and improve the negative symptoms in schizophrenia. These compounds, which have a considerable affinity for α_2_-adrenoceptors, would block α_2_-autoreceptors on noradrenergic neurons, activate noradrenergic activity and eventually produce a co-release of NE and DA in the PFC. This mechanism may also explain the enhanced efficacy of typical antipsychotics by add-on therapy with α_2_-adrenoceptors antagonists (Wadenberg et al., 2007; Hecht and Landy, 2012).

In conclusion, the finding that DA released by pharmacologically activated dopaminergic neurons may not be detected in the mPFC by *in vivo* microdialysis raises the important question of whether DA levels measured by microdialysis in the mPFC after stimuli such as feeding, drinking, sex, novelty, handling, foot-shock, etc. (Horvitz, 2000; Phillips et al., 2004) may indeed represent DA released from noradrenergic terminals.

These considerations challenge for further research on the role DA from of noradrenergic source in the physiology and dysfunctions of the PFC and indicate noradrenergic neurons as an important target for interventions aimed at modifying extracellular DA level in the PFC.

## Data Availability Statement

The raw data supporting the conclusions of this article will be made available by the authors, without undue reservation.

## Ethics Statement

The animal study was reviewed and approved by Ministero della Salute, Direzione Generale della sanità animale e dei farmaci veterinari, Uff. 6, Roma, Italy - Aut. n. 611/2017-PR.

## Author Contributions

PD and GLG designed the study and wrote the manuscript. PD supervised and analyzed microdialysis experiments. MP supervised electrophysiology experiments and contributed to the writing of the manuscript. CS and MS performed and analyzed electrophysiology experiments. PS and GF performed noradrenergic lesion and microdialysis experiments. All authors contributed to the article and approved the submitted version.

## Funding

The research was founded by the “Guy Everett Laboratory”, by “FSC 2014–2020—Patto per lo Sviluppo della Regione Sardegna, Legge Regionale n. 7 del 7 agosto 2007 (Bando 2017)” and by “Progetti di Rilevante Interesse Nazionale” (PRIN) 2017 (2017YH3SXK).

## Conflict of Interest

The authors declare that the research was conducted in the absence of any commercial or financial relationships that could be construed as a potential conflict of interest.
